# Supplementary material for: Natural hyperbolicity of hexagonal boron nitride in the deep ultraviolet
Source: Nat Commun. 2026 Feb 17;17:2869. doi: 10.1038/s41467-026-69536-4 (PMC13022243; doi:10.1038/s41467-026-69536-4)
Supplement: Supplementary file 1 — Supplementary Information [file 41467_2026_69536_MOESM1_ESM.pdf]

Supplementary Information

Natural Hyperbolicity of Hexagonal Boron Nitride in the Deep Ultraviolet

Bongjun Choi[1], Jason Lynch[1], Wangleong Chen[2], Seong-Joon Jeon[3,4], Hyungseob Cho[3,4], Kyungmin Yang[1], Jonghwan Kim[3,4,5]*, Nader Engheta[1,2,6,7]*, Deep Jariwala[1,2]*

[1]Department of Electrical and Systems Engineering, University of Pennsylvania, Philadelphia, Pennsylvania 19104, United States

[2]Department of Materials Science and Engineering, University of Pennsylvania, Philadelphia, Pennsylvania 19104, United States

[3]Department of Materials Science and Engineering, Pohang University of Science and Technology, Pohang, Republic of Korea.

[4]Center for van der Waals Quantum Solids, Institute for Basic Science (IBS), Pohang, Republic of Korea

[5]Department of Physics, Pohang University of Science and Technology, Pohang, Republic of Korea.

[6]Department of Bioengineering, University of Pennsylvania, Philadelphia, Pennsylvania 19104, United States

[7]Department of Physics and Astronomy, University of Pennsylvania, Philadelphia, Pennsylvania, 19104, United States

* Corresponding authors: dmj@seas.upenn.edu, engheta@seas.upenn.edu, jonghwankim@postech.ac.kr

The supporting information file includes:

Supplementary Figure 1. Simulated reflectance depending on the substrate.
Supplementary Figure 2. Map data of Ψ and Δ.
Supplementary Figure 3. Ellipsometry measurement results.
Supplementary Figure 4. Absorption coefficient of hBN.
Supplementary Figure 5. Determination of hBN thickness
Supplementary Figure 6. Simulated absorbance from monolayer hBN.
Supplementary Figure 7. Comparison of birefringence.
Supplementary Figure 8. The highly reflective plateau of hBN in the DUV regime.
Supplementary Figure 9. Isofrequency surface outside of the hyperbolic regime.
Supplementary Figure 10. Isofrequency surface of the hyperbolic regime.
Supplementary Figure 11. Simulated partial LDOS and Purcell factor
Supplementary Figure 12. Simulated electric profile from dipole.
Supplementary Figure 13. Excitation of high-k mode in the far field.
Supplementary Figure 14. Simulated electric profile in hyperbolic regime.
Supplementary Figure 15. Propagation distance and lifetime of HEP.
Supplementary Note 1. Analysis of imaging spectroscopic ellipsometry data
Supplementary Table 1. Anisotropic, complex refractive index of hBN
Supplementary References

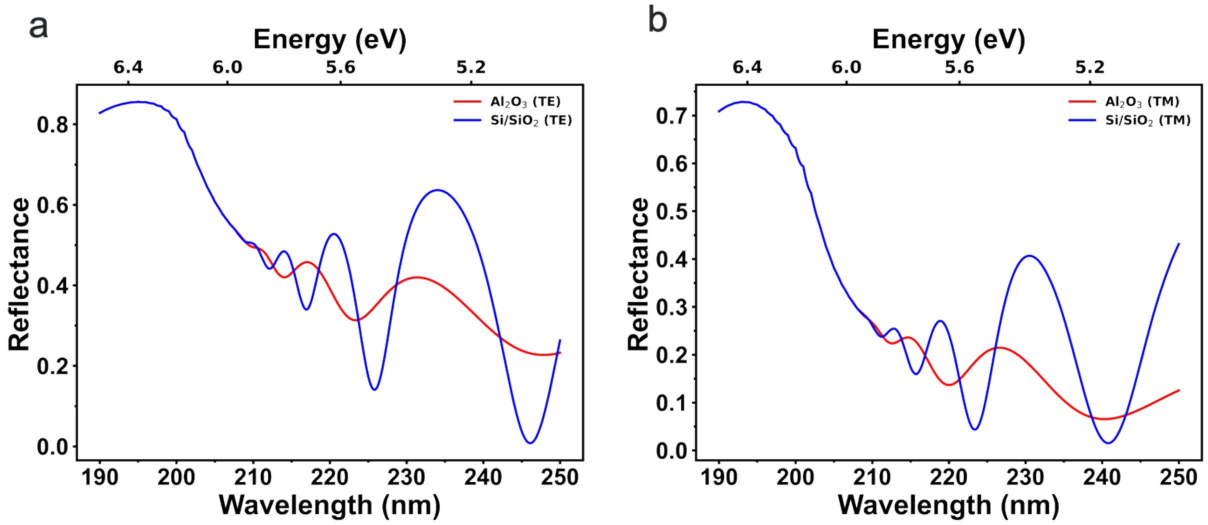

Supplementary Figure 1. Simulated reflectance spectra for 150 nm of hexagonal boron nitride (hBN) on Al_2O_3 and on Si/SiO_2 (300 nm) substrates at a 45 degree incidence angle for both (a) transverse-electric (TE) and (b) transverse-magnetic (TM) polarizations. The reflectance signal is notably weaker in the 210–250 nm range due to the strong absorption of the Si substrate.

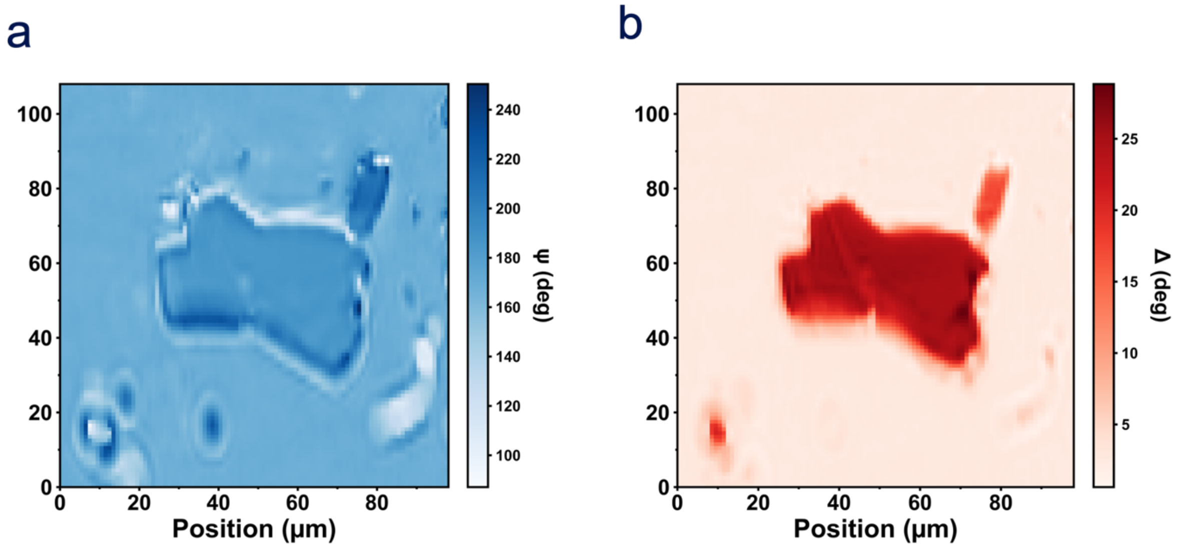

Supplementary Figure 2. Two-dimensional (2D) map data at 250 nm of (a) Ψ (the amplitude ratio) and (b) Δ (the phase difference) value from hBN using imaging spectroscopic ellipsometry (ISE). ISE allows simultaneous measurement of all pixels inside the area of interest (AOI). Each pixel ~ 1 x 1 μm contains the Ψ and Δ information.

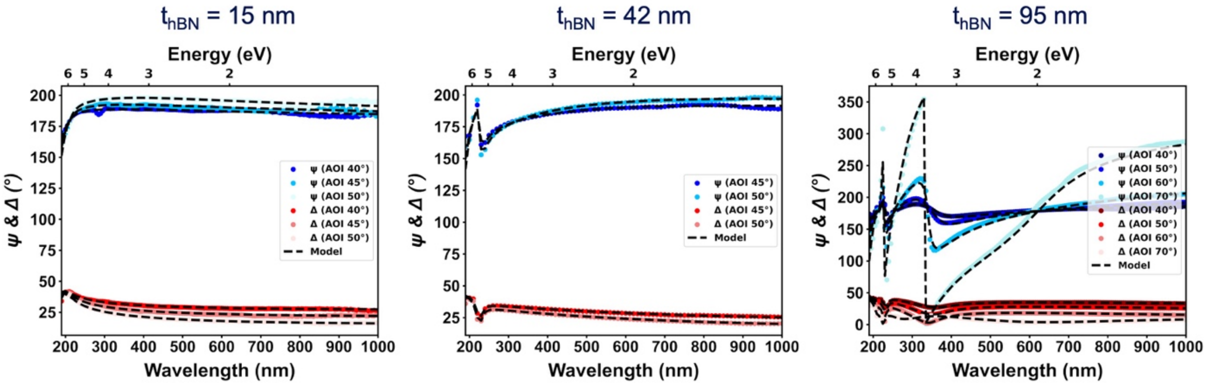

Supplementary Figure 3. Measured Ψ and Δ values for hBN films with varying thicknesses (15, 42, and 95 nm) and incident angles (40°~70°), overlaid with fitted curves based on a multi-Lorentz oscillator model. hBN was treated as a uniaxial material with independently fitted in-plane and out-of-plane components[1], demonstrating high fidelity of the optical model.

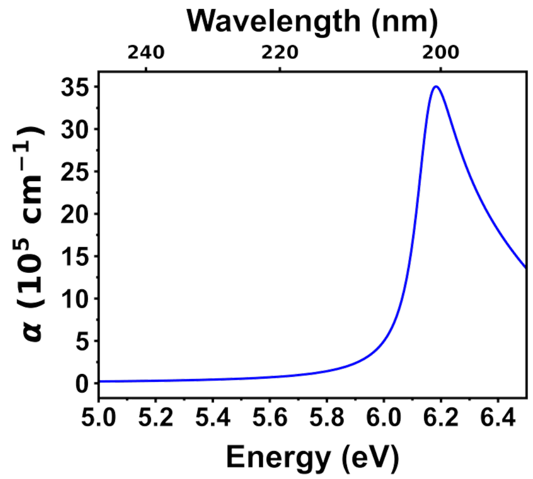

Supplementary Figure 4. Absorption coefficient ($\alpha = 4\pi k/\lambda$) of hBN in in-plane directions as a function of photon energy (wavelength), showing a significant absorption coefficient due to the strong exciton oscillator strength.

Supplementary Note 1. Analysis of imaging spectroscopic ellipsometry data

In ellipsometry measurement, the p- (transverse magnetic (TM)) and s- (transverse electric (TE)) polarized light are incident on the sample, measuring the polarization state of reflected light. Once the p- and s-polarizations of light hit the sample, each polarization of light shows different changes in amplitude and phase, and ellipsometry measures two parameters, Ψ and Δ, which represent the difference of amplitude and phase given by Eq. (1), where r_p and r_s are the Fresnel coefficients[1].

$$\rho = \frac{r_p}{r_s} = \tan\psi \; exp(i\Delta) \tag{1}$$

Ellipsometry is a model-based technique that requires two models to analyze the measured ISE data: the geometric structure of the sample and the dielectric function model. The geometric structure refers to the stacking sequence of the sample, including layer thicknesses. In order to increase the signal to noise ratio (SNR) in the DUV regime, we choose Al_2O_3 as a substrate due to its wide bandgap, and transferred the exfoliated hBN crystals. Thus, geometric structure is defined as an air / hBN (t nm) / Al_2O_3 (Substrate). Dielectric function model can describe the optical response, and especially electron transitions and exciton resonances are widely modelled using Lorentz oscillator model given by Eq. (2), where the resonance energy is E_0, f is the oscillator strength, and a damping factor is Γ.

$$\varepsilon_{Lorentz}(E) = \frac{fE_0\Gamma}{E_0^2 - E^2 - i\Gamma E} \tag{2}$$

If there are multiple oscillators in the systems, the system can be described by the sum of Lorentz oscillator model described in Eq. (3), where ε_∞ is a background permittivity accounting for high-energy electronic transitions that lie outside the measurable spectral range.

$$\varepsilon(E) = \varepsilon_\infty + \sum_i \varepsilon^i_{Lorentz}(E) \tag{3}$$

Since hBN has an anisotropy along z axis (uniaxial material), the dielectric tensor of hBN is represented by Eq. (4) since $\varepsilon_{xx} = \varepsilon_{yy}$.

$$\varepsilon = \begin{bmatrix} \varepsilon_{xx} & 0 & 0 \\ 0 & \varepsilon_{xx} & 0 \\ 0 & 0 & \varepsilon_{zz} \end{bmatrix} \tag{4}$$

Thus, ε_{xx} (in-plane permittivity) and ε_{zz} (out-of-plane permittivity) should be modelled respectively. Following previous literature[2-4], we model both in-plane and out-of-plane permittivity using multiple Lorentz oscillators with resonance energies of approximately 6.14, 6.82, and 14.97 eV for the in-plane component and 6.05 eV for the out-of-plane component within a uniaxial dielectric model, which accurately reproduces the experimental data, as shown in Supplementary Figure 2.

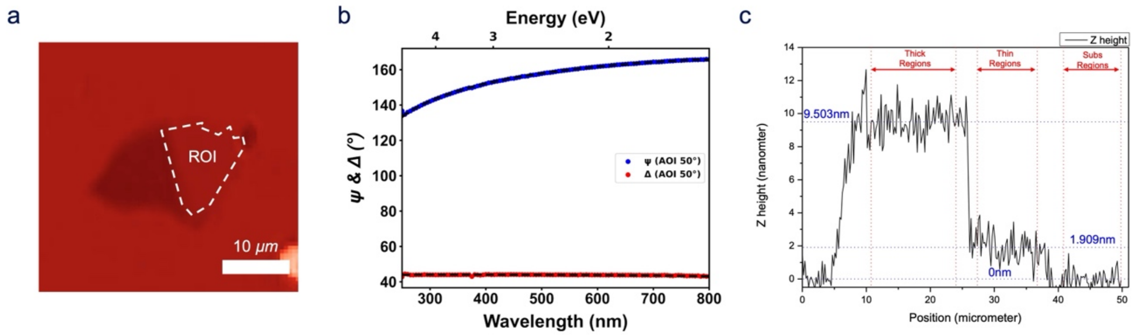

Supplementary Figure 5. Determination of hBN thickness using imaging spectroscopic ellipsometry (ISE). (a) Mapped Δ values from thin hBN. (b) Fitted curves based on hBN refractive indices, indicating a hBN thickness of ~1.7 nm (5 layers). (c) Thickness determination of hBN using atomic force microscopy (AFM) measurements, showing a consistent trend with the ellipsometry results.

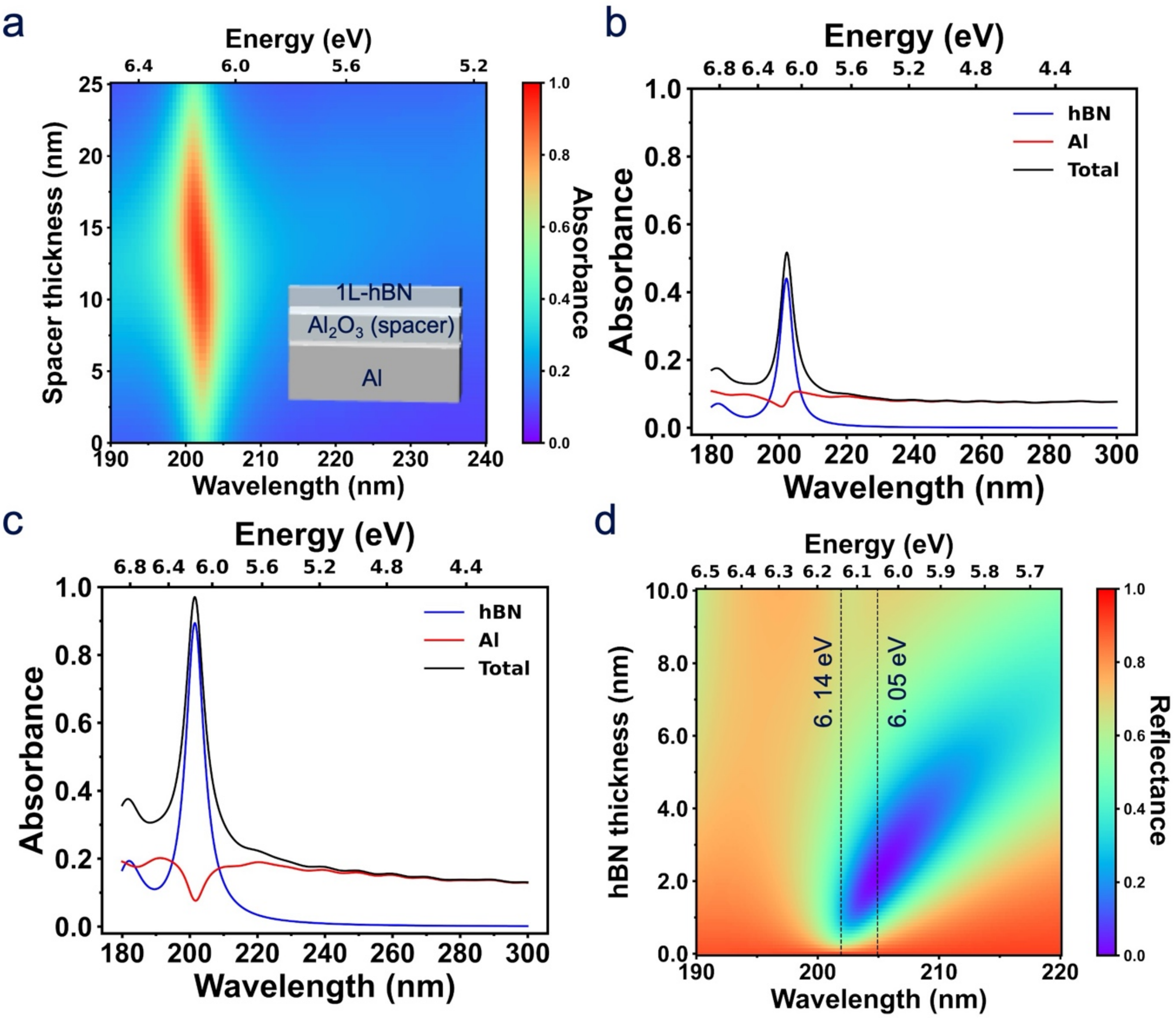

Supplementary Figure 6. (a) Simulated absorbance spectrum using transfer matrix method (TMM)[5-7]calculation in a 1L-hBN/Al_2O_3/Al heterostructure as a function of Al_2O_3 spacer thickness, demonstrating the high absorbance from only a monolayer hBN layer due to strong exciton resonance. Note that we assume that monolayer hBN

has the same refractive index as that of bulk for the calculation. Layer resolved absorbance spectrum depending on the presence and absence of the spacer structure: (b) 1L-hBN/Al (100 nm) and (c) 1L-hBN/Al_2O_3 (13.5 nm) /Al (100 nm). Even a monolayer of hBN on the Al substrate can absorb a significant amount of light. In addition, once an appropriate thickness of Al_2O_3 is added, absorbance can reach approximately unity absorption from a monolayer of hBN. Layer-resolved absorbance calculation shows that this significant amount of absorption mostly comes from the hBN monolayer for both (b) and (c) structures, indicating the strong exciton strength in hBN. (d) Simulated reflectance in a hBN/Al structure as a function of hBN thickness. As hBN approaches the monolayer limit, the excitonic peak is expected to appear near 6.14 eV. This behavior shows that the exciton resonance gradually red-shifts as the thickness increases.

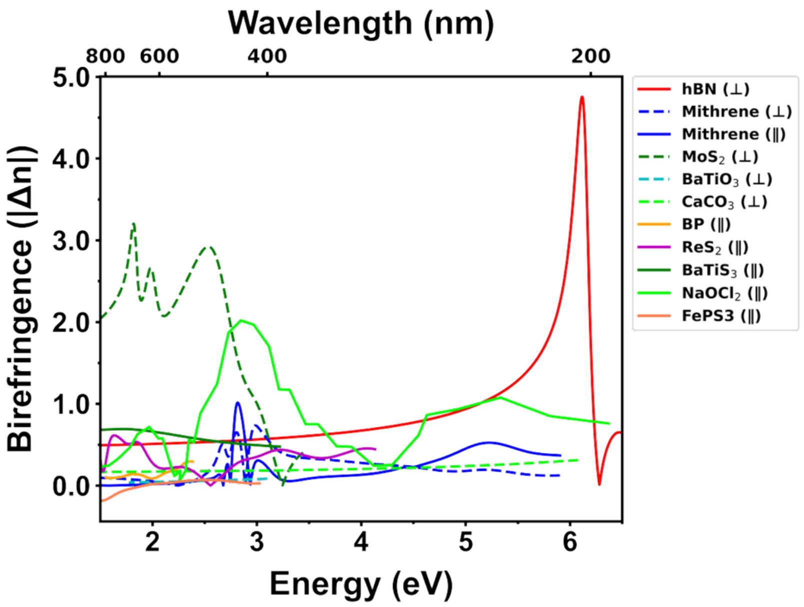

Supplementary Figure 7. Comparison of the in-plane and out-of-plane birefringence magnitude as a function of wavelength for various birefringent crystals[8-16], including hBN. hBN exhibits exceptionally large birefringence spanning the visible to the deep ultraviolet (DUV) range. Notably, it shows significant birefringence in the DUV regime, surpassing recently reported $NaOCl_2$, an uncommon and remarkable property.

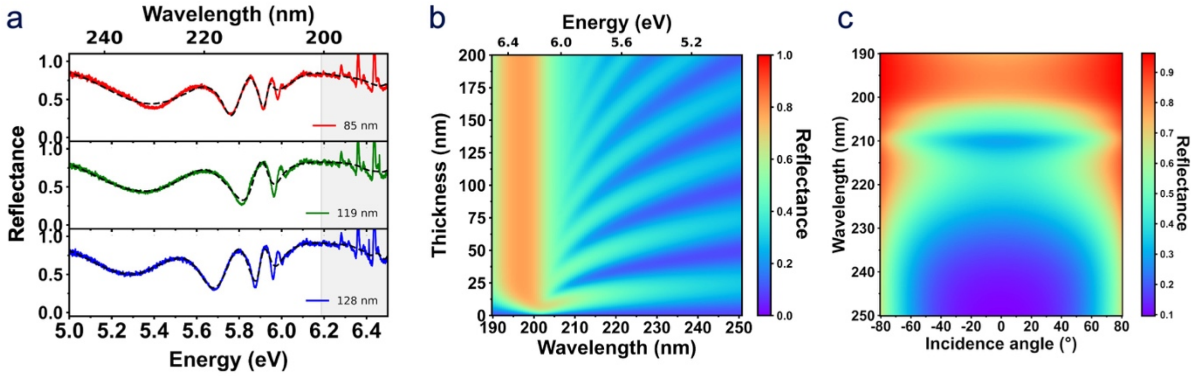

Supplementary Figure 8. (a) The measured reflectance spectrum with hBN on Al_2O_3 substrate reveals the highly reflective plateau (grey-shaded region), exhibiting high reflectance across multiple hBN thicknesses (85, 119, and 128 nm). The spikes observed around 190 nm are attributed to experimental artifacts, and the black dashed line indicates the smoothed profile generated using the UnivariateSpline function in SciPy. Simulated reflectance spectrum using TMM as a function of wavelength /thickness/incidence angle, showing the highly reflective plateau of hBN in the DUV regime in good agreement with experimental results in (a). (b) The thickness of hBN was changed on the Al_2O_3 substrate. As the hBN thickness approaches the optically thin limit, the highly reflective plateau changes to a single resonance. On the other hand, the thick enough hBN (> 25 nm), which is in the strong response regime, shows high reflectance. (c) Calculated reflectance as a function of the incidence angle of hBN (50nm) on the Al_2O_3 substrate structure, indicating a highly reflective nature in a wide range of incidence angles.

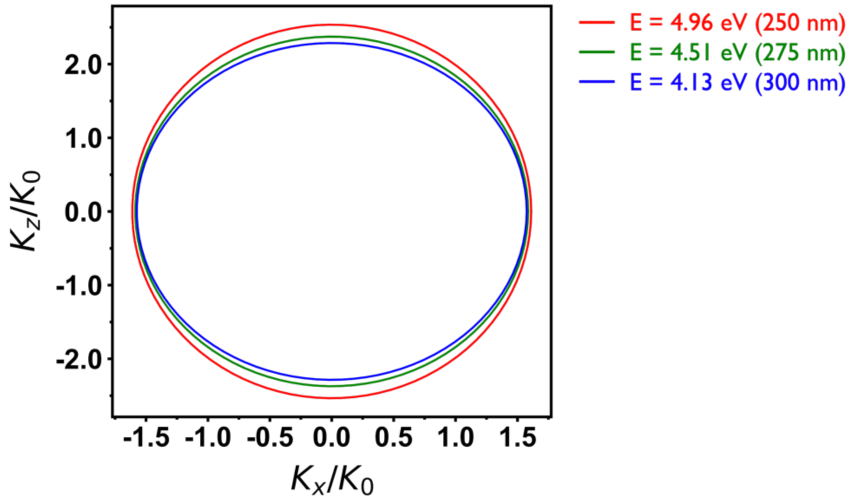

Supplementary Figure 9. Isofrequency surface in various photon energies (wavelengths) of hBN outside of the hyperbolic regime, showing the elliptical dispersion.

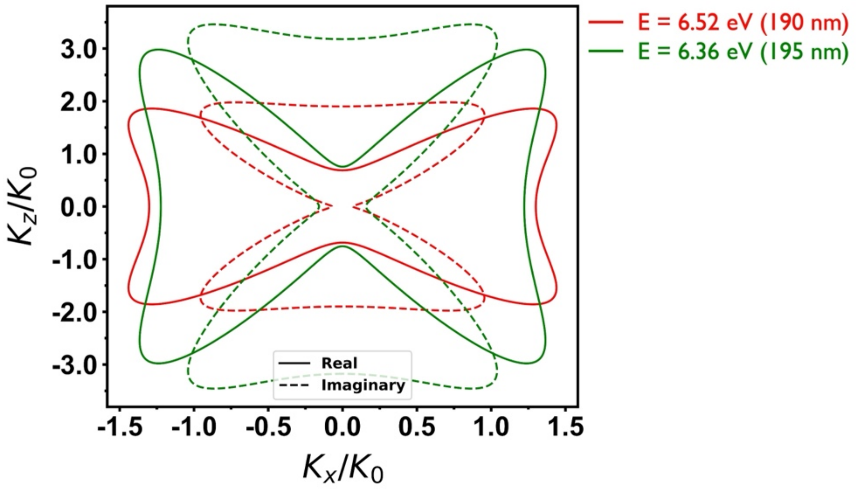

Supplementary Figure 10. Isofrequency surface in various photon energies (wavelengths) of hBN in the hyperbolic regime, showing the potential hyperbolic dispersion.

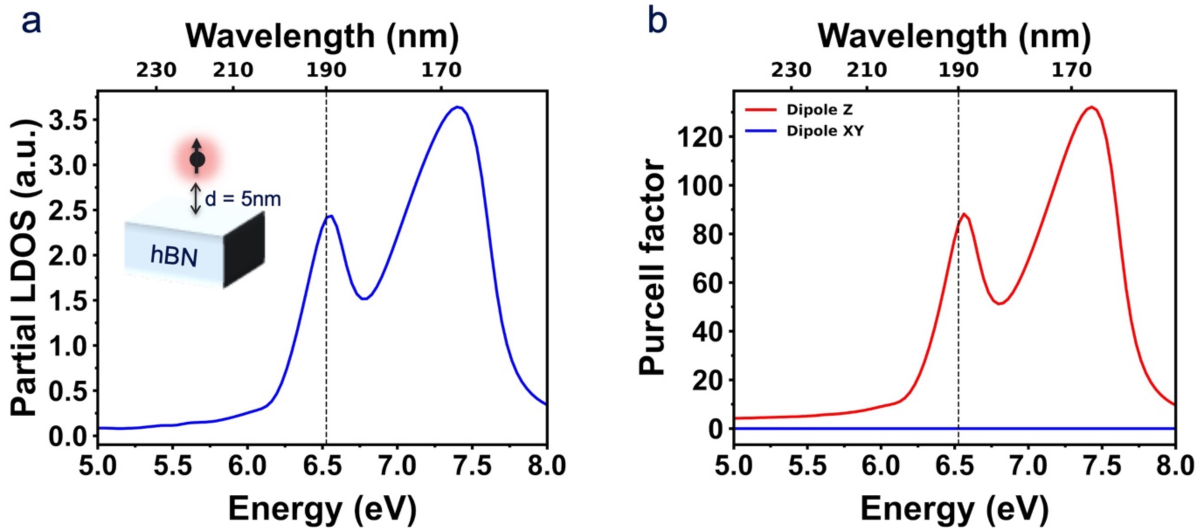

Supplementary Figure 11. (a) Calculated partial local density of states (LDOS) using finite-difference time-domain (FDTD) simulations. The dipole along the z-axis is located 5 nm above the hBN, as illustrated in the inset. The partial LDOS can be calculated from the imaginary part of the Green's function, demonstrating the larger partial LDOS in the hyperbolic regime[17]. (b) Simulated Purcell factor depending on the dipole orientation under the same conditions in (a). When the dipole is oriented along the z direction, the significant Purcell factor can be achieved, consistent with the partial LDOS calculation, as the Purcell factor is proportional to the LDOS. In contrast, an in-plane-oriented dipole does not contribute to emission enhancement, highlighting the critical role of hyperbolic dispersion in boosting the Purcell factor in hBN. In addition, a notable Purcell factor can be achieved around the epsilon near-zero (ENZ) point[18,19].

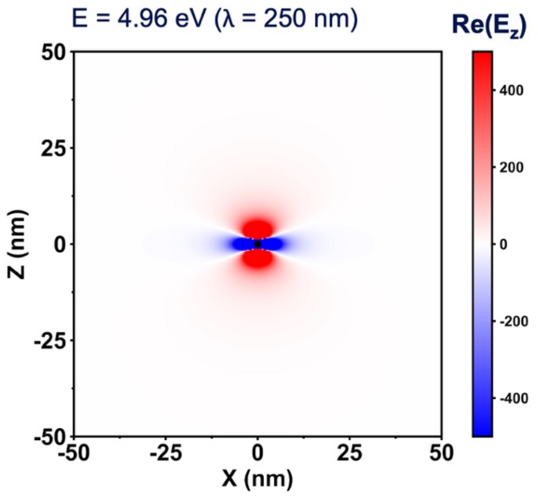

Supplementary Figure 12. Numerically simulated near-field distribution obtained from FDTD simulations shows the wave launched by a point dipole oriented along the Z-axis, outside the hyperbolic regime, in the XZ plane, exhibiting non-directional propagation behavior.

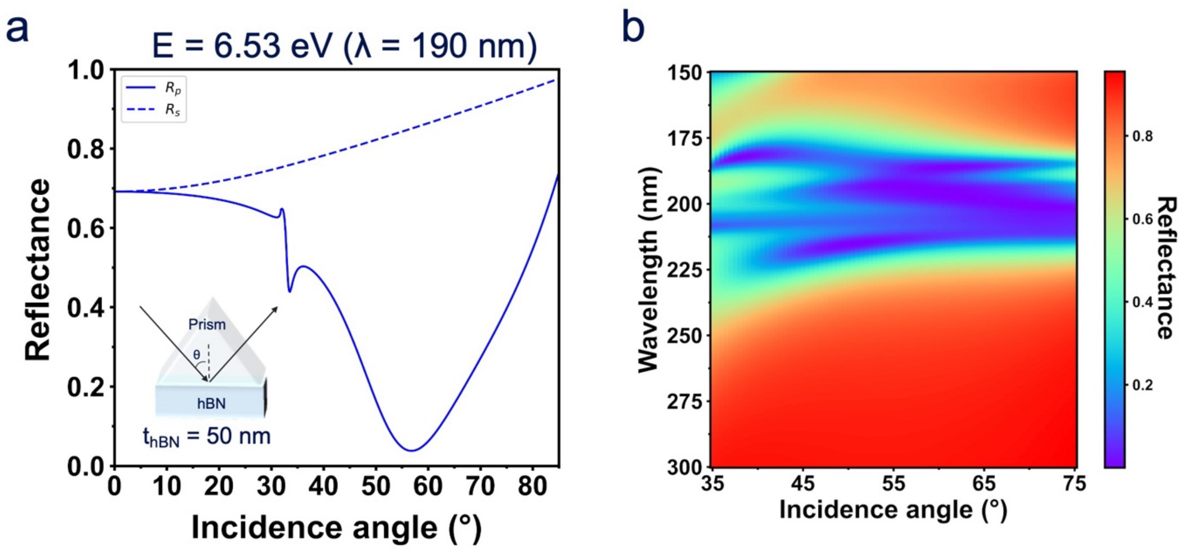

Supplementary Figure 13. (a) The calculated reflectance spectrum of 50 nm of hBN as a function of incidence angle at E = 6.53 eV (λ = 190 nm), depending on the polarization of incidence light (s (TE) and p (TM) polarization). Otto configuration is adapted using a high-index prism (Al_2O_3) to launch the highly confined high-k mode[20]. TE polarized light shows high reflectance associated with the hyperbolicity in hBN. In contrast, the pronounced dip appears around a 30° incidence angle in the TM polarized light incidence due to the coupling with the high-k mode[20]. Furthermore, Brewster's angle is observed around a 60° incidence angle, consistent with theoretical predictions calculated from the dielectric constants of the media. (b) The simulated reflectance spectrum as a function of incidence angle and wavelength, indicating a clear high-k wave launch in the hyperbolic window.

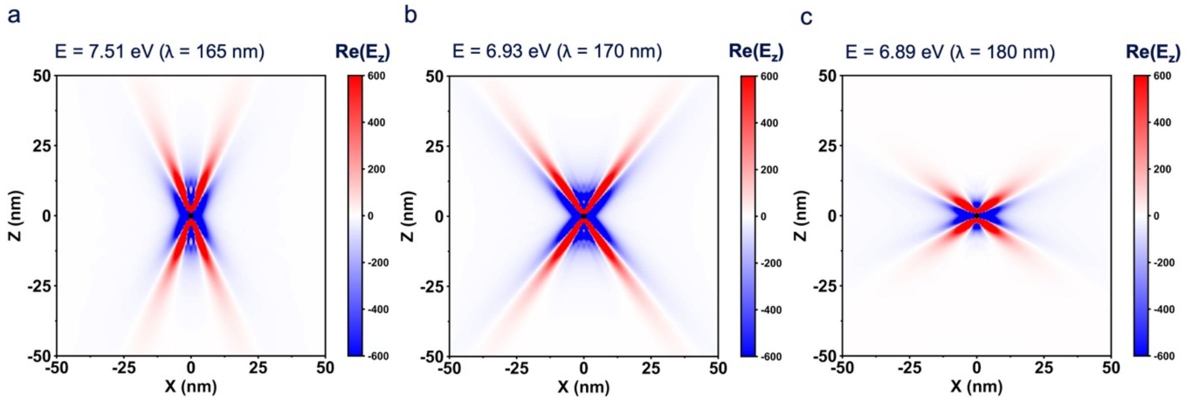

Supplementary Figure 14. Numerically simulated near-field wave propagation obtained from FDTD simulations as a function of photon energies (wavelengths) (a) 7.51 eV (λ = 165 nm), (b) 6.93 eV (λ = 170 nm), and (c) 6.89 eV (λ = 180 nm), showing highly directional propagation depending on the photon energies (wavelengths) in hyperbolic regime.

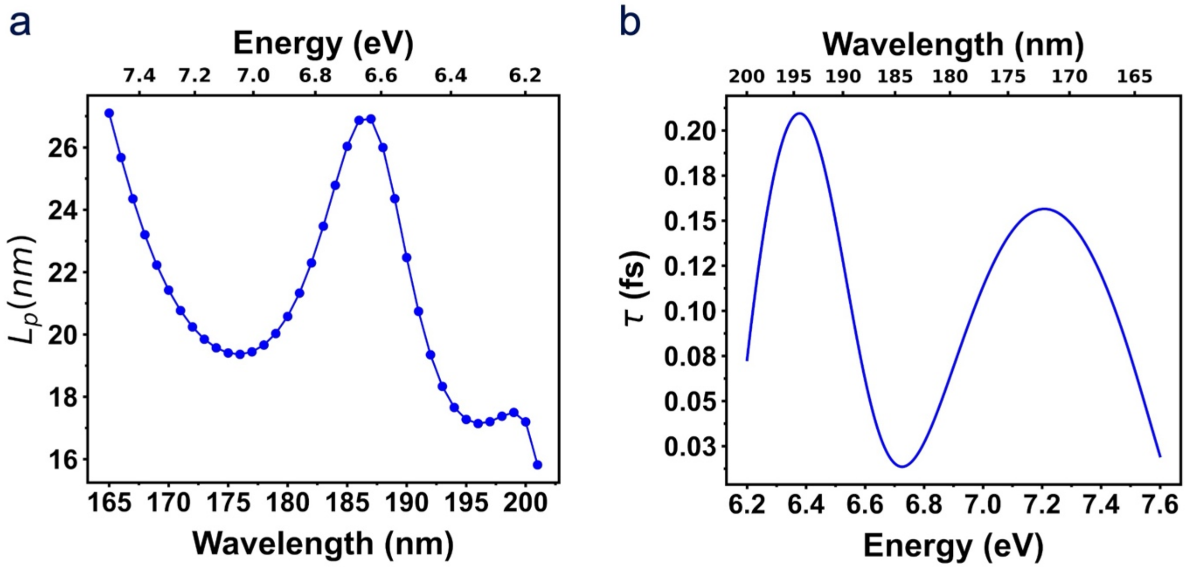

Supplementary Figure 15. (a) The propagation length of hyperbolic exciton polaritons (HEP) along the direction corresponding to the maximum real part of the wavevector in the hyperbolic regime[21]. (b) Calculated lifetime of HEP. The quality factor is approximately estimated as $Q = Re(k_{HEP})/Im(k_{HEP})$[22].

Supplementary Table 1. Anisotropic, complex refractive indices of hBN

wavelength (nm)	$n_{in\text{-}plane}$ (n_o)	$k_{in\text{-}plane}$ (k_o)	$n_{out\text{-}of\text{-}plane}$ (n_e)	$k_{out\text{-}of\text{-}plane}$ (k_e)
190	0.6866	1.8992	1.3006	0.0704
191	0.6398	2.1088	1.2873	0.0814
192	0.6234	2.3385	1.2728	0.0948
193	0.6355	2.5894	1.2571	0.1114
194	0.6774	2.8663	1.2403	0.1321
195	0.7552	3.1769	1.2229	0.1582
196	0.882	3.5321	1.2057	0.1912
197	1.0838	3.9453	1.1904	0.233
198	1.4114	4.429	1.1801	0.2854
199	1.9646	4.9751	1.1797	0.3494
200	2.9206	5.4731	1.196	0.4237
201	4.3979	5.4884	1.2363	0.5027
202	5.7732	4.4482	1.3058	0.5758
203	6.1308	2.9839	1.4032	0.6285
204	5.8395	1.9483	1.5183	0.6467
205	5.4229	1.3385	1.6326	0.6244
206	5.0454	0.9747	1.7269	0.5681
207	4.7314	0.745	1.7908	0.4935
208	4.4735	0.5916	1.8255	0.4165
209	4.2602	0.484	1.838	0.3469
210	4.0815	0.4055	1.8366	0.2882
211	3.9298	0.3463	1.8273	0.2405
212	3.7996	0.3005	1.8141	0.2022
213	3.6865	0.2642	1.7994	0.1716
214	3.5873	0.2349	1.7846	0.1471
215	3.4996	0.2108	1.7702	0.1272
216	3.4214	0.1907	1.7567	0.111
217	3.3512	0.1738	1.7441	0.0976
218	3.2879	0.1593	1.7325	0.0866
219	3.2303	0.1469	1.7219	0.0773
220	3.1779	0.1361	1.7121	0.0694
221	3.1298	0.1266	1.7031	0.0627
222	3.0855	0.1183	1.6949	0.0569
223	3.0447	0.1109	1.6873	0.052
224	3.0068	0.1043	1.6803	0.0476
225	2.9716	0.0984	1.6738	0.0438
226	2.9388	0.0931	1.6678	0.0405
227	2.9081	0.0883	1.6623	0.0376
228	2.8794	0.0839	1.6571	0.0349

229	2.8524	0.08	1.6523	0.0326
230	2.827	0.0763	1.6478	0.0305
231	2.8031	0.073	1.6436	0.0286
232	2.7805	0.0699	1.6397	0.0269
233	2.7591	0.067	1.636	0.0253
234	2.7389	0.0644	1.6326	0.0239
235	2.7196	0.062	1.6293	0.0226
236	2.7013	0.0597	1.6263	0.0215
237	2.6839	0.0576	1.6234	0.0204
238	2.6674	0.0556	1.6206	0.0194
239	2.6515	0.0537	1.618	0.0185
240	2.6364	0.052	1.6156	0.0176
241	2.622	0.0504	1.6133	0.0168
242	2.6081	0.0488	1.611	0.0161
243	2.5949	0.0474	1.6089	0.0154
244	2.5822	0.046	1.6069	0.0148
245	2.5699	0.0447	1.605	0.0142
246	2.5582	0.0435	1.6032	0.0137
247	2.5469	0.0423	1.6014	0.0131
248	2.5361	0.0412	1.5997	0.0127
249	2.5256	0.0402	1.5981	0.0122
250	2.5155	0.0392	1.5966	0.0118
251	2.5058	0.0383	1.5951	0.0114
252	2.4964	0.0374	1.5937	0.011
253	2.4874	0.0365	1.5924	0.0106
254	2.4786	0.0357	1.5911	0.0103
255	2.4702	0.0349	1.5898	0.01
256	2.462	0.0341	1.5886	0.0097
257	2.454	0.0334	1.5875	0.0094
258	2.4464	0.0327	1.5863	0.0091
259	2.4389	0.0321	1.5853	0.0088
260	2.4317	0.0314	1.5842	0.0086
261	2.4247	0.0308	1.5832	0.0084
262	2.4179	0.0302	1.5822	0.0081
263	2.4113	0.0297	1.5813	0.0079
264	2.4049	0.0291	1.5804	0.0077
265	2.3987	0.0286	1.5795	0.0075
266	2.3926	0.0281	1.5787	0.0073
267	2.3867	0.0276	1.5778	0.0072
268	2.381	0.0272	1.577	0.007
269	2.3754	0.0267	1.5763	0.0068
270	2.37	0.0263	1.5755	0.0067

271	2.3647	0.0259	1.5748	0.0065
272	2.3596	0.0254	1.5741	0.0064
273	2.3546	0.025	1.5734	0.0062
274	2.3497	0.0247	1.5727	0.0061
275	2.3449	0.0243	1.5721	0.006
276	2.3403	0.0239	1.5714	0.0058
277	2.3357	0.0236	1.5708	0.0057
278	2.3313	0.0233	1.5702	0.0056
279	2.3269	0.0229	1.5696	0.0055
280	2.3227	0.0226	1.5691	0.0054
281	2.3186	0.0223	1.5685	0.0053
282	2.3146	0.022	1.568	0.0052
283	2.3106	0.0217	1.5675	0.0051
284	2.3068	0.0214	1.567	0.005
285	2.303	0.0211	1.5665	0.0049
286	2.2993	0.0209	1.566	0.0048
287	2.2957	0.0206	1.5655	0.0047
288	2.2921	0.0204	1.565	0.0047
289	2.2887	0.0201	1.5646	0.0046
290	2.2853	0.0199	1.5641	0.0045
291	2.282	0.0196	1.5637	0.0044
292	2.2787	0.0194	1.5633	0.0043
293	2.2756	0.0192	1.5629	0.0043
294	2.2724	0.019	1.5625	0.0042
295	2.2694	0.0188	1.5621	0.0041
296	2.2664	0.0186	1.5617	0.0041
297	2.2635	0.0183	1.5613	0.004
298	2.2606	0.0181	1.561	0.004
299	2.2578	0.018	1.5606	0.0039
300	2.255	0.0178	1.5602	0.0038
301	2.2523	0.0176	1.5599	0.0038
302	2.2496	0.0174	1.5596	0.0037
303	2.247	0.0172	1.5592	0.0037
304	2.2444	0.0171	1.5589	0.0036
305	2.2419	0.0169	1.5586	0.0036
306	2.2394	0.0167	1.5583	0.0035
307	2.237	0.0166	1.558	0.0035
308	2.2346	0.0164	1.5577	0.0034
309	2.2322	0.0162	1.5574	0.0034
310	2.2299	0.0161	1.5571	0.0033
311	2.2277	0.0159	1.5568	0.0033
312	2.2254	0.0158	1.5565	0.0033

313	2.2232	0.0157	1.5563	0.0032
314	2.2211	0.0155	1.556	0.0032
315	2.219	0.0154	1.5557	0.0031
316	2.2169	0.0152	1.5555	0.0031
317	2.2148	0.0151	1.5552	0.0031
318	2.2128	0.015	1.555	0.003
319	2.2108	0.0148	1.5547	0.003
320	2.2089	0.0147	1.5545	0.003
321	2.207	0.0146	1.5543	0.0029
322	2.2051	0.0145	1.554	0.0029
323	2.2032	0.0144	1.5538	0.0029
324	2.2014	0.0142	1.5536	0.0028
325	2.1996	0.0141	1.5534	0.0028
326	2.1978	0.014	1.5532	0.0028
327	2.1961	0.0139	1.5529	0.0027
328	2.1944	0.0138	1.5527	0.0027
329	2.1927	0.0137	1.5525	0.0027
330	2.191	0.0136	1.5523	0.0026
331	2.1894	0.0135	1.5521	0.0026
332	2.1877	0.0134	1.5519	0.0026
333	2.1861	0.0133	1.5517	0.0026
334	2.1846	0.0132	1.5516	0.0025
335	2.183	0.0131	1.5514	0.0025
336	2.1815	0.013	1.5512	0.0025
337	2.18	0.0129	1.551	0.0025
338	2.1785	0.0128	1.5508	0.0024
339	2.1771	0.0127	1.5507	0.0024
340	2.1756	0.0126	1.5505	0.0024
341	2.1742	0.0125	1.5503	0.0024
342	2.1728	0.0125	1.5502	0.0024
343	2.1714	0.0124	1.55	0.0023
344	2.17	0.0123	1.5498	0.0023
345	2.1687	0.0122	1.5497	0.0023
346	2.1674	0.0121	1.5495	0.0023
347	2.1661	0.012	1.5494	0.0022
348	2.1648	0.012	1.5492	0.0022
349	2.1635	0.0119	1.5491	0.0022
350	2.1622	0.0118	1.5489	0.0022
351	2.161	0.0117	1.5488	0.0022
352	2.1598	0.0117	1.5486	0.0022
353	2.1586	0.0116	1.5485	0.0021
354	2.1574	0.0115	1.5483	0.0021

355	2.1562	0.0115	1.5482	0.0021
356	2.155	0.0114	1.5481	0.0021
357	2.1539	0.0113	1.5479	0.0021
358	2.1528	0.0112	1.5478	0.002
359	2.1516	0.0112	1.5477	0.002
360	2.1505	0.0111	1.5475	0.002
361	2.1495	0.011	1.5474	0.002
362	2.1484	0.011	1.5473	0.002
363	2.1473	0.0109	1.5472	0.002
364	2.1463	0.0109	1.547	0.002
365	2.1452	0.0108	1.5469	0.0019
366	2.1442	0.0107	1.5468	0.0019
367	2.1432	0.0107	1.5467	0.0019
368	2.1422	0.0106	1.5466	0.0019
369	2.1412	0.0105	1.5465	0.0019
370	2.1402	0.0105	1.5463	0.0019
371	2.1393	0.0104	1.5462	0.0019
372	2.1383	0.0104	1.5461	0.0018
373	2.1374	0.0103	1.546	0.0018
374	2.1364	0.0103	1.5459	0.0018
375	2.1355	0.0102	1.5458	0.0018
376	2.1346	0.0102	1.5457	0.0018
377	2.1337	0.0101	1.5456	0.0018
378	2.1328	0.01	1.5455	0.0018
379	2.1319	0.01	1.5454	0.0017
380	2.131	0.0099	1.5453	0.0017
381	2.1302	0.0099	1.5452	0.0017
382	2.1293	0.0098	1.5451	0.0017
383	2.1285	0.0098	1.545	0.0017
384	2.1277	0.0097	1.5449	0.0017
385	2.1268	0.0097	1.5448	0.0017
386	2.126	0.0096	1.5447	0.0017
387	2.1252	0.0096	1.5446	0.0017
388	2.1244	0.0095	1.5445	0.0016
389	2.1236	0.0095	1.5444	0.0016
390	2.1229	0.0095	1.5444	0.0016
391	2.1221	0.0094	1.5443	0.0016
392	2.1213	0.0094	1.5442	0.0016
393	2.1206	0.0093	1.5441	0.0016
394	2.1198	0.0093	1.544	0.0016
395	2.1191	0.0092	1.5439	0.0016
396	2.1184	0.0092	1.5439	0.0016

397	2.1176	0.0091	1.5438	0.0016
398	2.1169	0.0091	1.5437	0.0015
399	2.1162	0.0091	1.5436	0.0015
400	2.1155	0.009	1.5435	0.0015
401	2.1148	0.009	1.5435	0.0015
402	2.1141	0.0089	1.5434	0.0015
403	2.1134	0.0089	1.5433	0.0015
404	2.1128	0.0089	1.5432	0.0015
405	2.1121	0.0088	1.5431	0.0015
406	2.1114	0.0088	1.5431	0.0015
407	2.1108	0.0087	1.543	0.0015
408	2.1101	0.0087	1.5429	0.0015
409	2.1095	0.0087	1.5429	0.0014
410	2.1089	0.0086	1.5428	0.0014
411	2.1082	0.0086	1.5427	0.0014
412	2.1076	0.0086	1.5426	0.0014
413	2.107	0.0085	1.5426	0.0014
414	2.1064	0.0085	1.5425	0.0014
415	2.1058	0.0084	1.5424	0.0014
416	2.1052	0.0084	1.5424	0.0014
417	2.1046	0.0084	1.5423	0.0014
418	2.104	0.0083	1.5422	0.0014
419	2.1034	0.0083	1.5422	0.0014
420	2.1029	0.0083	1.5421	0.0014
421	2.1023	0.0082	1.5421	0.0014
422	2.1017	0.0082	1.542	0.0014
423	2.1012	0.0082	1.5419	0.0013
424	2.1006	0.0081	1.5419	0.0013
425	2.1001	0.0081	1.5418	0.0013
426	2.0995	0.0081	1.5417	0.0013
427	2.099	0.008	1.5417	0.0013
428	2.0985	0.008	1.5416	0.0013
429	2.0979	0.008	1.5416	0.0013
430	2.0974	0.0079	1.5415	0.0013
431	2.0969	0.0079	1.5415	0.0013
432	2.0964	0.0079	1.5414	0.0013
433	2.0959	0.0079	1.5413	0.0013
434	2.0954	0.0078	1.5413	0.0013
435	2.0949	0.0078	1.5412	0.0013
436	2.0944	0.0078	1.5412	0.0013
437	2.0939	0.0077	1.5411	0.0013
438	2.0934	0.0077	1.5411	0.0012

439	2.0929	0.0077	1.541	0.0012
440	2.0924	0.0076	1.541	0.0012
441	2.0919	0.0076	1.5409	0.0012
442	2.0915	0.0076	1.5409	0.0012
443	2.091	0.0076	1.5408	0.0012
444	2.0905	0.0075	1.5408	0.0012
445	2.0901	0.0075	1.5407	0.0012
446	2.0896	0.0075	1.5407	0.0012
447	2.0892	0.0075	1.5406	0.0012
448	2.0887	0.0074	1.5406	0.0012
449	2.0883	0.0074	1.5405	0.0012
450	2.0878	0.0074	1.5405	0.0012
451	2.0874	0.0073	1.5404	0.0012
452	2.087	0.0073	1.5404	0.0012
453	2.0866	0.0073	1.5403	0.0012
454	2.0861	0.0073	1.5403	0.0012
455	2.0857	0.0072	1.5402	0.0012
456	2.0853	0.0072	1.5402	0.0011
457	2.0849	0.0072	1.5401	0.0011
458	2.0845	0.0072	1.5401	0.0011
459	2.0841	0.0071	1.54	0.0011
460	2.0837	0.0071	1.54	0.0011
461	2.0833	0.0071	1.54	0.0011
462	2.0829	0.0071	1.5399	0.0011
463	2.0825	0.007	1.5399	0.0011
464	2.0821	0.007	1.5398	0.0011
465	2.0817	0.007	1.5398	0.0011
466	2.0813	0.007	1.5397	0.0011
467	2.0809	0.007	1.5397	0.0011
468	2.0805	0.0069	1.5397	0.0011
469	2.0802	0.0069	1.5396	0.0011
470	2.0798	0.0069	1.5396	0.0011
471	2.0794	0.0069	1.5395	0.0011
472	2.0791	0.0068	1.5395	0.0011
473	2.0787	0.0068	1.5395	0.0011
474	2.0783	0.0068	1.5394	0.0011
475	2.078	0.0068	1.5394	0.0011
476	2.0776	0.0068	1.5393	0.0011
477	2.0773	0.0067	1.5393	0.0011
478	2.0769	0.0067	1.5393	0.001
479	2.0766	0.0067	1.5392	0.001
480	2.0762	0.0067	1.5392	0.001

481	2.0759	0.0066	1.5392	0.001
482	2.0755	0.0066	1.5391	0.001
483	2.0752	0.0066	1.5391	0.001
484	2.0749	0.0066	1.5391	0.001
485	2.0745	0.0066	1.539	0.001
486	2.0742	0.0065	1.539	0.001
487	2.0739	0.0065	1.5389	0.001
488	2.0736	0.0065	1.5389	0.001
489	2.0732	0.0065	1.5389	0.001
490	2.0729	0.0065	1.5388	0.001
491	2.0726	0.0064	1.5388	0.001
492	2.0723	0.0064	1.5388	0.001
493	2.072	0.0064	1.5387	0.001
494	2.0717	0.0064	1.5387	0.001
495	2.0714	0.0064	1.5387	0.001
496	2.0711	0.0063	1.5386	0.001
497	2.0708	0.0063	1.5386	0.001
498	2.0705	0.0063	1.5386	0.001
499	2.0702	0.0063	1.5385	0.001
500	2.0699	0.0063	1.5385	0.001
501	2.0696	0.0063	1.5385	0.001
502	2.0693	0.0062	1.5384	0.001
503	2.069	0.0062	1.5384	0.001
504	2.0687	0.0062	1.5384	0.001
505	2.0684	0.0062	1.5384	0.0009
506	2.0681	0.0062	1.5383	0.0009
507	2.0678	0.0061	1.5383	0.0009
508	2.0676	0.0061	1.5383	0.0009
509	2.0673	0.0061	1.5382	0.0009
510	2.067	0.0061	1.5382	0.0009
511	2.0667	0.0061	1.5382	0.0009
512	2.0665	0.0061	1.5381	0.0009
513	2.0662	0.006	1.5381	0.0009
514	2.0659	0.006	1.5381	0.0009
515	2.0657	0.006	1.5381	0.0009
516	2.0654	0.006	1.538	0.0009
517	2.0651	0.006	1.538	0.0009
518	2.0649	0.006	1.538	0.0009
519	2.0646	0.0059	1.5379	0.0009
520	2.0644	0.0059	1.5379	0.0009
521	2.0641	0.0059	1.5379	0.0009
522	2.0638	0.0059	1.5379	0.0009

523	2.0636	0.0059	1.5378	0.0009
524	2.0633	0.0059	1.5378	0.0009
525	2.0631	0.0059	1.5378	0.0009
526	2.0629	0.0058	1.5378	0.0009
527	2.0626	0.0058	1.5377	0.0009
528	2.0624	0.0058	1.5377	0.0009
529	2.0621	0.0058	1.5377	0.0009
530	2.0619	0.0058	1.5377	0.0009
531	2.0616	0.0058	1.5376	0.0009
532	2.0614	0.0057	1.5376	0.0009
533	2.0612	0.0057	1.5376	0.0009
534	2.0609	0.0057	1.5376	0.0009
535	2.0607	0.0057	1.5375	0.0009
536	2.0605	0.0057	1.5375	0.0009
537	2.0602	0.0057	1.5375	0.0009
538	2.06	0.0057	1.5375	0.0008
539	2.0598	0.0056	1.5374	0.0008
540	2.0596	0.0056	1.5374	0.0008
541	2.0593	0.0056	1.5374	0.0008
542	2.0591	0.0056	1.5374	0.0008
543	2.0589	0.0056	1.5373	0.0008
544	2.0587	0.0056	1.5373	0.0008
545	2.0585	0.0056	1.5373	0.0008
546	2.0582	0.0055	1.5373	0.0008
547	2.058	0.0055	1.5372	0.0008
548	2.0578	0.0055	1.5372	0.0008
549	2.0576	0.0055	1.5372	0.0008
550	2.0574	0.0055	1.5372	0.0008
551	2.0572	0.0055	1.5372	0.0008
552	2.057	0.0055	1.5371	0.0008
553	2.0568	0.0054	1.5371	0.0008
554	2.0566	0.0054	1.5371	0.0008
555	2.0564	0.0054	1.5371	0.0008
556	2.0562	0.0054	1.537	0.0008
557	2.056	0.0054	1.537	0.0008
558	2.0558	0.0054	1.537	0.0008
559	2.0556	0.0054	1.537	0.0008
560	2.0554	0.0054	1.537	0.0008
561	2.0552	0.0053	1.5369	0.0008
562	2.055	0.0053	1.5369	0.0008
563	2.0548	0.0053	1.5369	0.0008
564	2.0546	0.0053	1.5369	0.0008

565	2.0544	0.0053	1.5369	0.0008
566	2.0542	0.0053	1.5368	0.0008
567	2.054	0.0053	1.5368	0.0008
568	2.0538	0.0053	1.5368	0.0008
569	2.0536	0.0052	1.5368	0.0008
570	2.0534	0.0052	1.5368	0.0008
571	2.0533	0.0052	1.5367	0.0008
572	2.0531	0.0052	1.5367	0.0008
573	2.0529	0.0052	1.5367	0.0008
574	2.0527	0.0052	1.5367	0.0008
575	2.0525	0.0052	1.5367	0.0008
576	2.0524	0.0052	1.5366	0.0008
577	2.0522	0.0051	1.5366	0.0008
578	2.052	0.0051	1.5366	0.0008
579	2.0518	0.0051	1.5366	0.0008
580	2.0516	0.0051	1.5366	0.0008
581	2.0515	0.0051	1.5366	0.0008
582	2.0513	0.0051	1.5365	0.0007
583	2.0511	0.0051	1.5365	0.0007
584	2.051	0.0051	1.5365	0.0007
585	2.0508	0.005	1.5365	0.0007
586	2.0506	0.005	1.5365	0.0007
587	2.0505	0.005	1.5364	0.0007
588	2.0503	0.005	1.5364	0.0007
589	2.0501	0.005	1.5364	0.0007
590	2.05	0.005	1.5364	0.0007
591	2.0498	0.005	1.5364	0.0007
592	2.0496	0.005	1.5364	0.0007
593	2.0495	0.005	1.5363	0.0007
594	2.0493	0.0049	1.5363	0.0007
595	2.0491	0.0049	1.5363	0.0007
596	2.049	0.0049	1.5363	0.0007
597	2.0488	0.0049	1.5363	0.0007
598	2.0487	0.0049	1.5363	0.0007
599	2.0485	0.0049	1.5362	0.0007
600	2.0484	0.0049	1.5362	0.0007
601	2.0482	0.0049	1.5362	0.0007
602	2.048	0.0049	1.5362	0.0007
603	2.0479	0.0049	1.5362	0.0007
604	2.0477	0.0048	1.5362	0.0007
605	2.0476	0.0048	1.5361	0.0007
606	2.0474	0.0048	1.5361	0.0007

607	2.0473	0.0048	1.5361	0.0007
608	2.0471	0.0048	1.5361	0.0007
609	2.047	0.0048	1.5361	0.0007
610	2.0468	0.0048	1.5361	0.0007
611	2.0467	0.0048	1.536	0.0007
612	2.0466	0.0048	1.536	0.0007
613	2.0464	0.0048	1.536	0.0007
614	2.0463	0.0047	1.536	0.0007
615	2.0461	0.0047	1.536	0.0007
616	2.046	0.0047	1.536	0.0007
617	2.0458	0.0047	1.536	0.0007
618	2.0457	0.0047	1.5359	0.0007
619	2.0456	0.0047	1.5359	0.0007
620	2.0454	0.0047	1.5359	0.0007
621	2.0453	0.0047	1.5359	0.0007
622	2.0451	0.0047	1.5359	0.0007
623	2.045	0.0047	1.5359	0.0007
624	2.0449	0.0046	1.5359	0.0007
625	2.0447	0.0046	1.5358	0.0007
626	2.0446	0.0046	1.5358	0.0007
627	2.0445	0.0046	1.5358	0.0007
628	2.0443	0.0046	1.5358	0.0007
629	2.0442	0.0046	1.5358	0.0007
630	2.0441	0.0046	1.5358	0.0007
631	2.0439	0.0046	1.5358	0.0007
632	2.0438	0.0046	1.5357	0.0007
633	2.0437	0.0046	1.5357	0.0007
634	2.0435	0.0046	1.5357	0.0007
635	2.0434	0.0045	1.5357	0.0007
636	2.0433	0.0045	1.5357	0.0007
637	2.0432	0.0045	1.5357	0.0007
638	2.043	0.0045	1.5357	0.0007
639	2.0429	0.0045	1.5357	0.0007
640	2.0428	0.0045	1.5356	0.0006
641	2.0427	0.0045	1.5356	0.0006
642	2.0425	0.0045	1.5356	0.0006
643	2.0424	0.0045	1.5356	0.0006
644	2.0423	0.0045	1.5356	0.0006
645	2.0422	0.0045	1.5356	0.0006
646	2.042	0.0044	1.5356	0.0006
647	2.0419	0.0044	1.5355	0.0006
648	2.0418	0.0044	1.5355	0.0006

649	2.0417	0.0044	1.5355	0.0006
650	2.0416	0.0044	1.5355	0.0006
651	2.0414	0.0044	1.5355	0.0006
652	2.0413	0.0044	1.5355	0.0006
653	2.0412	0.0044	1.5355	0.0006
654	2.0411	0.0044	1.5355	0.0006
655	2.041	0.0044	1.5355	0.0006
656	2.0409	0.0044	1.5354	0.0006
657	2.0407	0.0044	1.5354	0.0006
658	2.0406	0.0043	1.5354	0.0006
659	2.0405	0.0043	1.5354	0.0006
660	2.0404	0.0043	1.5354	0.0006
661	2.0403	0.0043	1.5354	0.0006
662	2.0402	0.0043	1.5354	0.0006
663	2.0401	0.0043	1.5354	0.0006
664	2.04	0.0043	1.5353	0.0006
665	2.0398	0.0043	1.5353	0.0006
666	2.0397	0.0043	1.5353	0.0006
667	2.0396	0.0043	1.5353	0.0006
668	2.0395	0.0043	1.5353	0.0006
669	2.0394	0.0043	1.5353	0.0006
670	2.0393	0.0042	1.5353	0.0006
671	2.0392	0.0042	1.5353	0.0006
672	2.0391	0.0042	1.5353	0.0006
673	2.039	0.0042	1.5352	0.0006
674	2.0389	0.0042	1.5352	0.0006
675	2.0388	0.0042	1.5352	0.0006
676	2.0387	0.0042	1.5352	0.0006
677	2.0386	0.0042	1.5352	0.0006
678	2.0385	0.0042	1.5352	0.0006
679	2.0384	0.0042	1.5352	0.0006
680	2.0383	0.0042	1.5352	0.0006
681	2.0382	0.0042	1.5352	0.0006
682	2.0381	0.0042	1.5351	0.0006
683	2.038	0.0042	1.5351	0.0006
684	2.0379	0.0041	1.5351	0.0006
685	2.0378	0.0041	1.5351	0.0006
686	2.0377	0.0041	1.5351	0.0006
687	2.0376	0.0041	1.5351	0.0006
688	2.0375	0.0041	1.5351	0.0006
689	2.0374	0.0041	1.5351	0.0006
690	2.0373	0.0041	1.5351	0.0006

691	2.0372	0.0041	1.5351	0.0006
692	2.0371	0.0041	1.535	0.0006
693	2.037	0.0041	1.535	0.0006
694	2.0369	0.0041	1.535	0.0006
695	2.0368	0.0041	1.535	0.0006
696	2.0367	0.0041	1.535	0.0006
697	2.0366	0.004	1.535	0.0006
698	2.0365	0.004	1.535	0.0006
699	2.0364	0.004	1.535	0.0006
700	2.0363	0.004	1.535	0.0006
701	2.0362	0.004	1.535	0.0006
702	2.0361	0.004	1.5349	0.0006
703	2.036	0.004	1.5349	0.0006
704	2.0359	0.004	1.5349	0.0006
705	2.0359	0.004	1.5349	0.0006
706	2.0358	0.004	1.5349	0.0006
707	2.0357	0.004	1.5349	0.0006
708	2.0356	0.004	1.5349	0.0006
709	2.0355	0.004	1.5349	0.0006
710	2.0354	0.004	1.5349	0.0006
711	2.0353	0.004	1.5349	0.0006
712	2.0352	0.0039	1.5349	0.0006
713	2.0351	0.0039	1.5348	0.0006
714	2.0351	0.0039	1.5348	0.0006
715	2.035	0.0039	1.5348	0.0006
716	2.0349	0.0039	1.5348	0.0006
717	2.0348	0.0039	1.5348	0.0006
718	2.0347	0.0039	1.5348	0.0006
719	2.0346	0.0039	1.5348	0.0006
720	2.0345	0.0039	1.5348	0.0006
721	2.0345	0.0039	1.5348	0.0005
722	2.0344	0.0039	1.5348	0.0005
723	2.0343	0.0039	1.5348	0.0005
724	2.0342	0.0039	1.5347	0.0005
725	2.0341	0.0039	1.5347	0.0005
726	2.034	0.0039	1.5347	0.0005
727	2.034	0.0038	1.5347	0.0005
728	2.0339	0.0038	1.5347	0.0005
729	2.0338	0.0038	1.5347	0.0005
730	2.0337	0.0038	1.5347	0.0005
731	2.0336	0.0038	1.5347	0.0005
732	2.0336	0.0038	1.5347	0.0005

733	2.0335	0.0038	1.5347	0.0005
734	2.0334	0.0038	1.5347	0.0005
735	2.0333	0.0038	1.5347	0.0005
736	2.0332	0.0038	1.5346	0.0005
737	2.0332	0.0038	1.5346	0.0005
738	2.0331	0.0038	1.5346	0.0005
739	2.033	0.0038	1.5346	0.0005
740	2.0329	0.0038	1.5346	0.0005
741	2.0329	0.0038	1.5346	0.0005
742	2.0328	0.0038	1.5346	0.0005
743	2.0327	0.0038	1.5346	0.0005
744	2.0326	0.0037	1.5346	0.0005
745	2.0325	0.0037	1.5346	0.0005
746	2.0325	0.0037	1.5346	0.0005
747	2.0324	0.0037	1.5346	0.0005
748	2.0323	0.0037	1.5346	0.0005
749	2.0322	0.0037	1.5345	0.0005
750	2.0322	0.0037	1.5345	0.0005
751	2.0321	0.0037	1.5345	0.0005
752	2.032	0.0037	1.5345	0.0005
753	2.032	0.0037	1.5345	0.0005
754	2.0319	0.0037	1.5345	0.0005
755	2.0318	0.0037	1.5345	0.0005
756	2.0317	0.0037	1.5345	0.0005
757	2.0317	0.0037	1.5345	0.0005
758	2.0316	0.0037	1.5345	0.0005
759	2.0315	0.0037	1.5345	0.0005
760	2.0315	0.0037	1.5345	0.0005
761	2.0314	0.0036	1.5345	0.0005
762	2.0313	0.0036	1.5344	0.0005
763	2.0312	0.0036	1.5344	0.0005
764	2.0312	0.0036	1.5344	0.0005
765	2.0311	0.0036	1.5344	0.0005
766	2.031	0.0036	1.5344	0.0005
767	2.031	0.0036	1.5344	0.0005
768	2.0309	0.0036	1.5344	0.0005
769	2.0308	0.0036	1.5344	0.0005
770	2.0308	0.0036	1.5344	0.0005
771	2.0307	0.0036	1.5344	0.0005
772	2.0306	0.0036	1.5344	0.0005
773	2.0306	0.0036	1.5344	0.0005
774	2.0305	0.0036	1.5344	0.0005

775	2.0304	0.0036	1.5344	0.0005
776	2.0304	0.0036	1.5344	0.0005
777	2.0303	0.0036	1.5343	0.0005
778	2.0302	0.0036	1.5343	0.0005
779	2.0302	0.0035	1.5343	0.0005
780	2.0301	0.0035	1.5343	0.0005
781	2.03	0.0035	1.5343	0.0005
782	2.03	0.0035	1.5343	0.0005
783	2.0299	0.0035	1.5343	0.0005
784	2.0298	0.0035	1.5343	0.0005
785	2.0298	0.0035	1.5343	0.0005
786	2.0297	0.0035	1.5343	0.0005
787	2.0296	0.0035	1.5343	0.0005
788	2.0296	0.0035	1.5343	0.0005
789	2.0295	0.0035	1.5343	0.0005
790	2.0295	0.0035	1.5343	0.0005
791	2.0294	0.0035	1.5343	0.0005
792	2.0293	0.0035	1.5342	0.0005
793	2.0293	0.0035	1.5342	0.0005
794	2.0292	0.0035	1.5342	0.0005
795	2.0291	0.0035	1.5342	0.0005
796	2.0291	0.0035	1.5342	0.0005
797	2.029	0.0035	1.5342	0.0005
798	2.029	0.0035	1.5342	0.0005
799	2.0289	0.0034	1.5342	0.0005
800	2.0288	0.0034	1.5342	0.0005
801	2.0288	0.0034	1.5342	0.0005
802	2.0287	0.0034	1.5342	0.0005
803	2.0287	0.0034	1.5342	0.0005
804	2.0286	0.0034	1.5342	0.0005
805	2.0285	0.0034	1.5342	0.0005
806	2.0285	0.0034	1.5342	0.0005
807	2.0284	0.0034	1.5342	0.0005
808	2.0284	0.0034	1.5341	0.0005
809	2.0283	0.0034	1.5341	0.0005
810	2.0283	0.0034	1.5341	0.0005
811	2.0282	0.0034	1.5341	0.0005
812	2.0281	0.0034	1.5341	0.0005
813	2.0281	0.0034	1.5341	0.0005
814	2.028	0.0034	1.5341	0.0005
815	2.028	0.0034	1.5341	0.0005
816	2.0279	0.0034	1.5341	0.0005

817	2.0279	0.0034	1.5341	0.0005
818	2.0278	0.0034	1.5341	0.0005
819	2.0277	0.0033	1.5341	0.0005
820	2.0277	0.0033	1.5341	0.0005
821	2.0276	0.0033	1.5341	0.0005
822	2.0276	0.0033	1.5341	0.0005
823	2.0275	0.0033	1.5341	0.0005
824	2.0275	0.0033	1.5341	0.0005
825	2.0274	0.0033	1.534	0.0005
826	2.0274	0.0033	1.534	0.0005
827	2.0273	0.0033	1.534	0.0005
828	2.0273	0.0033	1.534	0.0005
829	2.0272	0.0033	1.534	0.0005
830	2.0271	0.0033	1.534	0.0005
831	2.0271	0.0033	1.534	0.0005
832	2.027	0.0033	1.534	0.0005
833	2.027	0.0033	1.534	0.0005
834	2.0269	0.0033	1.534	0.0005
835	2.0269	0.0033	1.534	0.0005
836	2.0268	0.0033	1.534	0.0005
837	2.0268	0.0033	1.534	0.0005
838	2.0267	0.0033	1.534	0.0005
839	2.0267	0.0033	1.534	0.0005
840	2.0266	0.0033	1.534	0.0005
841	2.0266	0.0032	1.534	0.0004
842	2.0265	0.0032	1.534	0.0004
843	2.0265	0.0032	1.534	0.0004
844	2.0264	0.0032	1.5339	0.0004
845	2.0264	0.0032	1.5339	0.0004
846	2.0263	0.0032	1.5339	0.0004
847	2.0263	0.0032	1.5339	0.0004
848	2.0262	0.0032	1.5339	0.0004
849	2.0262	0.0032	1.5339	0.0004
850	2.0261	0.0032	1.5339	0.0004
851	2.0261	0.0032	1.5339	0.0004
852	2.026	0.0032	1.5339	0.0004
853	2.026	0.0032	1.5339	0.0004
854	2.0259	0.0032	1.5339	0.0004
855	2.0259	0.0032	1.5339	0.0004
856	2.0258	0.0032	1.5339	0.0004
857	2.0258	0.0032	1.5339	0.0004
858	2.0257	0.0032	1.5339	0.0004

859	2.0257	0.0032	1.5339	0.0004
860	2.0256	0.0032	1.5339	0.0004
861	2.0256	0.0032	1.5339	0.0004
862	2.0255	0.0032	1.5339	0.0004
863	2.0255	0.0032	1.5339	0.0004
864	2.0254	0.0032	1.5338	0.0004
865	2.0254	0.0031	1.5338	0.0004
866	2.0254	0.0031	1.5338	0.0004
867	2.0253	0.0031	1.5338	0.0004
868	2.0253	0.0031	1.5338	0.0004
869	2.0252	0.0031	1.5338	0.0004
870	2.0252	0.0031	1.5338	0.0004
871	2.0251	0.0031	1.5338	0.0004
872	2.0251	0.0031	1.5338	0.0004
873	2.025	0.0031	1.5338	0.0004
874	2.025	0.0031	1.5338	0.0004
875	2.0249	0.0031	1.5338	0.0004
876	2.0249	0.0031	1.5338	0.0004
877	2.0249	0.0031	1.5338	0.0004
878	2.0248	0.0031	1.5338	0.0004
879	2.0248	0.0031	1.5338	0.0004
880	2.0247	0.0031	1.5338	0.0004
881	2.0247	0.0031	1.5338	0.0004
882	2.0246	0.0031	1.5338	0.0004
883	2.0246	0.0031	1.5338	0.0004
884	2.0245	0.0031	1.5338	0.0004
885	2.0245	0.0031	1.5337	0.0004
886	2.0245	0.0031	1.5337	0.0004
887	2.0244	0.0031	1.5337	0.0004
888	2.0244	0.0031	1.5337	0.0004
889	2.0243	0.0031	1.5337	0.0004
890	2.0243	0.003	1.5337	0.0004
891	2.0242	0.003	1.5337	0.0004
892	2.0242	0.003	1.5337	0.0004
893	2.0242	0.003	1.5337	0.0004
894	2.0241	0.003	1.5337	0.0004
895	2.0241	0.003	1.5337	0.0004
896	2.024	0.003	1.5337	0.0004
897	2.024	0.003	1.5337	0.0004
898	2.0239	0.003	1.5337	0.0004
899	2.0239	0.003	1.5337	0.0004
900	2.0239	0.003	1.5337	0.0004

901	2.0238	0.003	1.5337	0.0004
902	2.0238	0.003	1.5337	0.0004
903	2.0237	0.003	1.5337	0.0004
904	2.0237	0.003	1.5337	0.0004
905	2.0237	0.003	1.5337	0.0004
906	2.0236	0.003	1.5337	0.0004
907	2.0236	0.003	1.5337	0.0004
908	2.0235	0.003	1.5337	0.0004
909	2.0235	0.003	1.5336	0.0004
910	2.0235	0.003	1.5336	0.0004
911	2.0234	0.003	1.5336	0.0004
912	2.0234	0.003	1.5336	0.0004
913	2.0233	0.003	1.5336	0.0004
914	2.0233	0.003	1.5336	0.0004
915	2.0233	0.003	1.5336	0.0004
916	2.0232	0.0029	1.5336	0.0004
917	2.0232	0.0029	1.5336	0.0004
918	2.0231	0.0029	1.5336	0.0004
919	2.0231	0.0029	1.5336	0.0004
920	2.0231	0.0029	1.5336	0.0004
921	2.023	0.0029	1.5336	0.0004
922	2.023	0.0029	1.5336	0.0004
923	2.023	0.0029	1.5336	0.0004
924	2.0229	0.0029	1.5336	0.0004
925	2.0229	0.0029	1.5336	0.0004
926	2.0228	0.0029	1.5336	0.0004
927	2.0228	0.0029	1.5336	0.0004
928	2.0228	0.0029	1.5336	0.0004
929	2.0227	0.0029	1.5336	0.0004
930	2.0227	0.0029	1.5336	0.0004
931	2.0227	0.0029	1.5336	0.0004
932	2.0226	0.0029	1.5336	0.0004
933	2.0226	0.0029	1.5336	0.0004
934	2.0225	0.0029	1.5335	0.0004
935	2.0225	0.0029	1.5335	0.0004
936	2.0225	0.0029	1.5335	0.0004
937	2.0224	0.0029	1.5335	0.0004
938	2.0224	0.0029	1.5335	0.0004
939	2.0224	0.0029	1.5335	0.0004
940	2.0223	0.0029	1.5335	0.0004
941	2.0223	0.0029	1.5335	0.0004
942	2.0223	0.0029	1.5335	0.0004

943	2.0222	0.0029	1.5335	0.0004
944	2.0222	0.0029	1.5335	0.0004
945	2.0222	0.0028	1.5335	0.0004
946	2.0221	0.0028	1.5335	0.0004
947	2.0221	0.0028	1.5335	0.0004
948	2.022	0.0028	1.5335	0.0004
949	2.022	0.0028	1.5335	0.0004
950	2.022	0.0028	1.5335	0.0004
951	2.0219	0.0028	1.5335	0.0004
952	2.0219	0.0028	1.5335	0.0004
953	2.0219	0.0028	1.5335	0.0004
954	2.0218	0.0028	1.5335	0.0004
955	2.0218	0.0028	1.5335	0.0004
956	2.0218	0.0028	1.5335	0.0004
957	2.0217	0.0028	1.5335	0.0004
958	2.0217	0.0028	1.5335	0.0004
959	2.0217	0.0028	1.5335	0.0004
960	2.0216	0.0028	1.5335	0.0004
961	2.0216	0.0028	1.5335	0.0004
962	2.0216	0.0028	1.5334	0.0004
963	2.0215	0.0028	1.5334	0.0004
964	2.0215	0.0028	1.5334	0.0004
965	2.0215	0.0028	1.5334	0.0004
966	2.0214	0.0028	1.5334	0.0004
967	2.0214	0.0028	1.5334	0.0004
968	2.0214	0.0028	1.5334	0.0004
969	2.0213	0.0028	1.5334	0.0004
970	2.0213	0.0028	1.5334	0.0004
971	2.0213	0.0028	1.5334	0.0004
972	2.0212	0.0028	1.5334	0.0004
973	2.0212	0.0028	1.5334	0.0004
974	2.0212	0.0028	1.5334	0.0004
975	2.0211	0.0028	1.5334	0.0004
976	2.0211	0.0027	1.5334	0.0004
977	2.0211	0.0027	1.5334	0.0004
978	2.021	0.0027	1.5334	0.0004
979	2.021	0.0027	1.5334	0.0004
980	2.021	0.0027	1.5334	0.0004
981	2.021	0.0027	1.5334	0.0004
982	2.0209	0.0027	1.5334	0.0004
983	2.0209	0.0027	1.5334	0.0004
984	2.0209	0.0027	1.5334	0.0004

985	2.0208	0.0027	1.5334	0.0004
986	2.0208	0.0027	1.5334	0.0004
987	2.0208	0.0027	1.5334	0.0004
988	2.0207	0.0027	1.5334	0.0004
989	2.0207	0.0027	1.5334	0.0004
990	2.0207	0.0027	1.5334	0.0004
991	2.0206	0.0027	1.5334	0.0004
992	2.0206	0.0027	1.5334	0.0004
993	2.0206	0.0027	1.5333	0.0004
994	2.0206	0.0027	1.5333	0.0004
995	2.0205	0.0027	1.5333	0.0004
996	2.0205	0.0027	1.5333	0.0004
997	2.0205	0.0027	1.5333	0.0004
998	2.0204	0.0027	1.5333	0.0004
999	2.0204	0.0027	1.5333	0.0004
1000	2.0204	0.0027	1.5333	0.0004

Supplementary References

1 Fujiwara, H. *Spectroscopic ellipsometry: principles and applications*. (John Wiley & Sons, 2007).

2 Arnaud, B., Lebègue, S., Rabiller, P. & Alouani, M. Huge excitonic effects in layered hexagonal boron nitride. *Physical review letters* **96**, 026402 (2006).

3 Artús, L. *et al.* Ellipsometry study of hexagonal boron nitride using synchrotron radiation: transparency window in the Far-UVC. *Advanced Photonics Research* **2**, 2000101 (2021).

4 Tarrio, C. & Schnatterly, S. Interband transitions, plasmons, and dispersion in hexagonal boron nitride. *Physical review B* **40**, 7852 (1989).

5 Pettersson, L. A., Roman, L. S. & Inganäs, O. Modeling photocurrent action spectra of photovoltaic devices based on organic thin films. *Journal of Applied Physics* **86**, 487-496 (1999).

6 Peumans, P., Yakimov, A. & Forrest, S. R. Small molecular weight organic thin-film photodetectors and solar cells. *Journal of Applied Physics* **93**, 3693-3723 (2003).

7 Passler, N. C. & Paarmann, A. Generalized 4× 4 matrix formalism for light propagation in anisotropic stratified media: study of surface phonon polaritons in polar dielectric heterostructures. *Journal of the Optical Society of America B* **34**, 2128-2139 (2017).

8 Choi, B. *et al.* Giant Optical Anisotropy in 2D Metal–Organic Chalcogenates. *ACS Nano* **18**, 25489-25498 (2024). https://doi.org:10.1021/acsnano.4c05043

9 Ermolaev, G. *et al.* Giant optical anisotropy in transition metal dichalcogenides for next-generation photonics. *Nature communications* **12**, 854 (2021).

10 Wemple, S. H., Didomenico, M. & Camlibel, I. Dielectric and optical properties of melt-grown BaTiO3. *Journal of Physics and Chemistry of Solids* **29**, 1797-1803 (1968). https://doi.org:https://doi.org/10.1016/0022-3697(68)90164-9

11 Ghosh, G. Dispersion-equation coefficients for the refractive index and birefringence of calcite and quartz crystals. *Optics communications* **163**, 95-102 (1999).

12 Yang, H. *et al.* Optical Waveplates Based on Birefringence of Anisotropic Two-Dimensional Layered Materials. *ACS Photonics* **4**, 3023-3030 (2017). https://doi.org:10.1021/acsphotonics.7b00507

13 Munkhbat, B., Wróbel, P., Antosiewicz, T. J. & Shegai, T. O. Optical Constants of Several Multilayer Transition Metal Dichalcogenides Measured by Spectroscopic Ellipsometry in the 300–1700 nm Range: High Index, Anisotropy, and Hyperbolicity. *ACS Photonics* **9**, 2398-2407 (2022). https://doi.org:10.1021/acsphotonics.2c00433

14 Niu, S. *et al.* Giant optical anisotropy in a quasi-one-dimensional crystal. *Nature Photonics* **12**, 392-396 (2018).

15 Guo, Q. *et al.* Colossal in-plane optical anisotropy in a two-dimensional van der Waals crystal. *Nature Photonics*, 1-6 (2024).

16 Zhang, H. *et al.* Cavity-enhanced linear dichroism in a van der Waals antiferromagnet. *Nature Photonics* **16**, 311-317 (2022).

17 Novotny, L. & Hecht, B. *Principles of nano-optics*. (Cambridge university press, 2012).

18 Chebykin, A., Orlov, A., Shalin, A., Poddubny, A. & Belov, P. Strong Purcell effect in anisotropic ε-near-zero metamaterials. *Physical Review B* **91**, 205126 (2015).

19 Bharadwaj, P. & Novotny, L. Spectral dependence of single molecule fluorescence enhancement. *Optics express* **15**, 14266-14274 (2007).

20 Maier, S. A. *Plasmonics: fundamentals and applications*. Vol. 1 (Springer, 2007).

21 Jackson, E., Tischler, J., Ratchford, D. & Ellis, C. The role of losses in determining hyperbolic material figures of merit. *Scientific Reports* **14**, 25156 (2024).

22 Giles, A. J. *et al.* Ultralow-loss polaritons in isotopically pure boron nitride. *Nature Materials* **17**, 134-139 (2018). https://doi.org:10.1038/nmat5047
